# Gender-common and gender-specific determinants of child dietary diversity in eight Asia Pacific countries

**DOI:** 10.7189/jogh.12.04058

**Published:** 2022-10-01

**Authors:** Huilin Li, Yunjeong Kim, Chulwoo Park, Minji Kang, Yunhee Kang

**Affiliations:** 1 Johns Hopkins University School of Education, Baltimore, Maryland, USA; 2 Johns Hopkins Bloomberg School of Public Health, Baltimore, Maryland, USA; 3 Department of Public Health and Recreation, San José State University, San Jose, California, USA; 4 Brain Korea 21 FOUR Education and Research Team for Sustainable Food & Nutrition, Department of Food and Nutrition, College of Human Ecology, Seoul National University, Seoul, Republic of Korea; 5 Department of International Health, Johns Hopkins Bloomberg School of Public Health, Baltimore, Maryland, USA

## Abstract

**Background:**

Optimal child feeding practices contribute to reducing child undernutrition in low- and middle-income countries. Minimum dietary diversity (MDD) is a key indicator of complementary feeding quality for children aged 6-23 months. We aimed to examine the gender-common and gender-specific factors associated with the failure to meet MDD in eight Asia Pacific countries.

**Methods:**

The study used data of children aged 6-23 months from the Demographic and Health Surveys (DHS) conducted in Afghanistan (n = 8410), Bangladesh (n = 2371), Nepal (n = 1478), Pakistan (n = 3490), Cambodia (n = 2182), Indonesia (n = 5133), Myanmar (n = 1379), and Timor-Leste (n = 2115). A total of 41 household, maternal, and child-level variables were examined for association with MDD using univariate and multivariable logistic regressions. All analyses accounted for the survey design and sampling weights.

**Results:**

Being aged 6-11 months, not receiving Vitamin A supplementation, low maternal education, belonging to a low wealth quintile, and having two or more young children in the household were factors related to the failure to meet MDD among both male and female children. Mothers’ not watching TV or not being exposed to media at least once a week, delivery at home, young age, and engagement to non-agricultural work were only significant risk factors among female children. Non-professional delivery assistance, unsafe disposal of children’s stool, tolerant attitudes towards domestic violence, and rural residence were significant factors only among male children.

**Conclusions:**

It is possible that male and female children in the region may consume food in various ways, because the factors for meeting MDD are not the same for different genders of children. It is advised to enhance dietary diversity in child nutrition programmes through gender-specific activities.

Consumption of diverse foods is essential for the healthy growth of children [1]. After being exclusively breastfed until six months of age, sufficient nutrients through complementary foods need to be supplied for children’s continuous growth [1]. Minimum dietary diversity (MDD) is one of the key indicators for measuring the quality of complementary feeding for children aged 6-23 months [2]. It is defined as the proportion of children 6-23 months of age who consume five or more of eight food groups (grains, roots and tubers, legumes and nuts, dairy products, eggs, flesh foods, vitamin A-rich fruits and vegetables, other fruits and vegetables, and breastmilk) [3]. The association between high dietary diversity and improved nutrition among children under five years of age has been reported in low- and middle-income countries [4-6]. A study using data from the Demographic and Health Surveys (DHS) from 11 countries reported a positive association between dietary diversity and height-for-age Z-scores for children aged 6-23 months [6].

Globally, only 28% of children aged 6-23 months met the MDD in 2020 [7]. Only 37% of South Asian children met the MDD, while those in Southeast Asia and the Pacific have achieved 43% [8]. South Asia has a higher prevalence of stunted children (32%) likely due to a lower proportion of MDD, whereas Southeast Asia had a lower prevalence of stunting (25%) in 2020 [8].

The nutritional status of children in the Asia Pacific region reflects different economic statuses, food systems, and affordability of diverse food options. The population of South Asia is rising rapidly, yet farm production is declining, and the cost of agricultural products and household poverty are increasing [9]. A study by Ryckman et al. [10] found a lack of diversity of affordable food sources in South Asia. For example, dark, leafy green vegetables are the only affordable iron, calcium, and zinc source in Bangladesh, India, and Pakistan. In Southeast Asia, however, the food system adapted to modern retail markets with urbanization, rising incomes, and transformation to commercial production [11]. Urbanization and retail market structure can increase the diversity of affordable food by driving income growth and decreasing the food price [12,13]. Nevertheless, poor child dietary diversity remains a concern among poor populations in countries with traditional dietary behaviour, which is largely dependent on staple grains and vegetables [14].

Food choice is a complex process influenced by individual, socio-economic, cultural, and environmental factors. There have been substantial efforts to determine the factors of child food consumption and dietary behaviours [15-17]. Studies in individual countries in the Asia Pacific region reported various factors predicting a child’s dietary diversity such as household wealth, living location, parental education, antenatal and postnatal care, employment type of household head, younger child age, younger maternal age, and exposure to media [18-23].

Gender-based differences in the child dietary patterns have been the interest of many previous studies [24-28]. Borooah et al. [24] found girls are more likely to be neglected than boys with respect to receiving nutritious diets in India. Ng et al. [15] found more events of insufficient complementary feeding among female children in Indonesia. Fledderjohann et al. [25] found that female children in India were breastfed for a shorter duration and had lower consumption of dairy food compared to male children. These differences can be explained by gender disparity in the intra-household food allocation for children, which is affected by cultural norms in society and women’s empowerment in households [25,29].

While there is some literature revealing children’s gender as a predictor of dietary quality, little is known about the gender-specific predictors of dietary diversity. This study examined gender-common and gender-specific determinants of the failure to meet MDD among young children in eight low- and middle-income countries in the Asia Pacific region while hypothesising that predictors of failing to meet MDD differ between male and female children in the region.

## METHODS

### Study design and participants

This study used the most recent DHS data for eight South and Southeast Asian countries: Afghanistan Demographic Health Survey (AfDHS) 2015 [30], Bangladesh Demographic Health Survey (BDHS) 2017-2018 [31], Nepal Demographic Health Survey (NDHS) 2016 [32], Pakistan Demographic Health Survey (PDHS) 2017–2018 [33], Cambodia Demographic Health Survey (CDHS) 2014 [34], Indonesia Demographic Health Survey (IDHS) 2017 [35], Myanmar Demographic Health Survey (MDHS) 2015–2016 [36], and Timor-Leste Demographic Health Survey (TLDHS) 2016 [37]. The survey participants were sampled based on a multi-stage stratified cluster sampling design. Only the data of children aged 6-23 months with available dietary information were used in the analyses. The final sample of respondents for South Asia included: 8410 in Afghanistan, 2436 in Bangladesh, 1478 in Nepal, and 3490 in Pakistan. In Southeast Asia, the number of respondents in the data analysis was as follows: 2182 in Cambodia, 5113 in Indonesia, 1379 in Myanmar, and 2115 in Timor-Leste.

### Dependent variable: Minimum Dietary Diversity (MDD)

The MDD has been universally used to measure the quality of dietary patterns among children aged 6-23 months old [2]. It is defined by the United Nations International Children’s Emergency Fund (UNICEF) standard guideline of MDD as the consumption of five or more food groups out of eight groups [3].

During DHS data collection, respondents were asked to report the type of food their child consumed in the last 24 hours. We constructed a child’s dietary diversity score ranging from 0 to 8 based on the child’s food consumption of eight food groups: 1) grains, roots, and tubers, 2) legumes and nuts, 3) dairy products, 4) eggs, 5) flesh foods, 6) vitamin A-rich fruits and vegetables, 7) other fruits and vegetables, and 8) breastmilk [3]. As the original DHS had more detailed food categories for fresh foods (ie, meat, organ meat, and fish) and grains (ie, bread, potatoes), we recategorized those items into the eight food groups above. Children who reported consuming at least one food item in the food group received one score for that food group. For breastfeeding, one score was given to the children whose mothers were practising breastfeeding at the time of data collection. Finally, the dietary diversity score was dichotomized by considering a total score over five as satisfying MDD [3].

### Independent variables

Potential factors of the failure to meet MDD were selected based on the conceptual framework of child dietary diversity in developing countries (Figure S1 in the **Online Supplementary Document**). A total of 41 variables were selected at household, maternal, and child levels.

Household-level characteristics included a total of nine variables: 1) residential area (rural or urban), 2) wealth quantiles, 3) sex of household head, 4) number of household members, 5) number of children under five in the household, 6) type of drinking water source, 7) type of sanitation facility, (8) proximity to water source, and 9) type of cooking fuel. In the original DHS data set, respondents were asked to report their drinking water sources and sanitation facility types using multiple categories. We applied World Health Organization (WHO) and UNICEF’s guidelines to classify the categories into improved or unimproved types [38].

Maternal characteristics comprised 22 variables: 1) age, 2) education, 3) occupation, 4) living with their partner or not, 5) BMI (kg/m2), 6) smoking status, 7) pregnancy, 8) number of antenatal clinic visits, 9) place of delivery, 10) type of delivery assistance, 11) postnatal check-up within two days after delivery, 12) disposal of child’s stool, 13) reading the newspaper at least once a week, 14) listening to the radio at least once a week, 15) watching TV at least once a week, 16) exposure to all three media sources – newspaper, radio, and TV – at least once a week, 17) overall attitude toward domestic violence, 18) decision-making on respondent’s health care, 19) decision making on large household purchases, 20) decision-making on visits to family or relatives, 21) decision-making on what to do with money husband earns, and 22) women’s empowerment.

The mother’s attitude toward domestic violence was assessed by asking mothers whether domestic violence would be justified in the following scenarios: a) if the wife goes out without telling the husband, b) if the wife neglects the children, c) if the wife argues with the husband, d) if the wife refuses to have sex with husband, and e) if the wife overcooks the food. If mothers answered that beating is unjustified in any of the scenarios, they were considered to have an intolerant attitude toward domestic violence. The variable for women’s empowerment was created using their attitude toward domestic violence and their participation in decision-making in health care, large purchases, family/relative visits, and the spending of the husband’s wage. We summed all five scores of women’s attitudes toward domestic violence and participation in decision-making to generate a total score ranging from 0 to 5. The total score was then dichotomized by treating a total score higher than 3 as high empowerment and others as low empowerment [38].

Lastly, child-level characteristics included a total of 10 variables as follows: 1) gender, 2) age (6-11 months vs 12-23 months), 3) birth order, 4) perceived birth size (small, average, and large), 5) episodes of diarrhoea, 6) cough, 7) fever in the past two weeks, 8) anaemia, 9) vitamin A supplementation in the past six months, and 10) iron supplement intake, such as pills, sprinkles, and syrup in the past seven days.

### Statistical analysis

Sampling design and survey weights of the DHS data were accounted for in all analyses. The weighted percentage of the household, maternal, and child characteristics were explored for each country. Weighted χ^2^ tests were conducted to compare MDD status between male and female children in the eight countries.

We conducted gender-stratified analyses to identify the factors associated with the failure to meet MDD. First, univariate logistic regressions were conducted to identify the characteristics significantly associated with the MDD status of male and female children separately (judged by 95% confidence interval (CI) of unadjusted odds ratios (ORs) excluding 1.00). Second, multivariable regressions were performed to calculate adjusted ORs for not meeting MDD. The multivariable regression models only included the variables that were significant in the univariate regressions. The variables with significant ORs in the multivariable logistic regressions for both males and females were gender-common factors of not meeting MDD. Likewise, the variables which were significant only among either male or female children were treated as male-specific or female-specific factors for not meeting MDD. These gender-stratified analyses were conducted separately for each country. A multivariable regression model was built for each country based on the covariates’ selection process using univariate regression. Finally, the heatmaps were created to present the risk factors descriptively for male and female children. All analyses were conducted using Stata 16 (StataCorp LP, College Station, TX, USA).

### Ethical approval

The survey protocol and questionnaires of the 2015 AfDHS were approved [30]. Ethical approval for the 2014 BDHS was given by the National Institute of Population Research and Training (NIPORT) of the Bangladesh Ministry of Health and Family Welfare [31]. The National Bioethics Committee of the Pakistan Health Research Council reviewed and approved the 2017-18 PDHS protocol [32]. The Nepal Health Research Council (NHRC) reviewed and approved the 2016 NDHS protocol [33]. The 2014 CDHS was approved by the National Ethics Committee for Health Research [34]. The 2017 IDHS passed the ethical review from the National Institute for Research and Development of the Indonesian Ministry of Health [35]. The 2015-16 MDHS protocol was reviewed and approved by the Ethics Review Committee on Medical Research Including Human Subjects in the Myanmar Ministry of Health and Sports' Department of Medical Research [36]. The previously mentioned DHS protocols were reviewed and approved by the ICF Institutional Review Board and Afghanistan's Ministry of Public Health. The questionnaires and survey protocol of the 2016 TLDHS was reviewed and approved by the ICF Institutional Review Board [37]. For all eight DHS data sets above, written informed consents were obtained from the survey respondents before data collection. The DHS data are publicly accessible and were made available upon our request to the DHS Program, ICF International.

## RESULTS

### Household, maternal, and child characteristics

Out of the eight countries included in the present study, Nepal had the highest proportion of households in an urban area (53.4%), while Pakistan had the lowest (13.7%). Most household heads in eight countries were male (range = 73.1%-99.0%) (**Table 1**). The proportion of households with five or fewer members was highest in Indonesia (60.9%) and lowest in Afghanistan (17.8%). The percentage of having two or more children under five years old was the highest in Afghanistan (80.3%). Most households used improved drinking water (61.6%-86.7%). More than half of the households in the study countries used improved toilet facilities (50.5%-72.6%), with the exception of those in Afghanistan (32.6%). Most households in Indonesia had a drinking water source in their house (84.4%), while fewer than half had it in Afghanistan (45.2%). Notably, most countries in the study had a low proportion of efficient cooking fuel usage (eg, 12.9% in Timor-Leste). However, Indonesian respondents reported high use of efficient cooking fuels (78.7%).

**Table 1:** Weighted percentage of child characteristics at household, maternal and individual level in eight countries in South Asia and Southeast Asia

Characteristics	Afghanistan (n = 8410)	Bangladesh (n = 2436)	Pakistan (n = 2181)	Nepal (n = 1478)	Cambodia (n = 2182)	Indonesia (n = 5113)	Myanmar (n = 1379)	Timor-Leste (n = 2115)
	Weighted %*	Weighted %*	Weighted %*	Weighted %*	Weighted %*	Weighted %*	Weighted %*	Weighted %*
**Household level**								
Rural resident	75.5	72.5	86.3	46.6	86.3	50.9	75.1	73.6
Female household head	1.0	13.4	22.4	26.9	22.4	9.3	14.0	11.0
Household members ≥6	82.2	46.6	50.7	56.6	50.7	39.1	49.1	69.1
2 or more children <5 y old living in the same household	80.3	69.7	44.6	48.8	44.6	31.0	40.7	67.3
Unimproved drinking water source†	32.5	13.3	12.4	13.0	-	38.4	22.0	21.0
Unimproved toilet facility‡	67.4	46.0	49.0	29.2	49.0	27.4	49.5	40.5
Time to get to water source ≥60 min	5.4	-	7.6	10.5	-	4.4	4.8	10.5
Inefficient cooking fuel	68.1	85.5	84.4	77.8	84.4	21.4	81.8	87.1
**Maternal level**								
Age of 15-24	36.7	44.6	34.6	51.6	34.6	23.0	25.2	27.9
No formal maternal education	79.9	7.4	12.9	30.2	12.9	0.8	15.3	22.4
Not working	87.4	57.3	31.5	45.6	31.5	55.9	42.0	65.9
Not living with partner	2.4	19.6	5.7	42.2	5.7	9.5	7.1	9.5
BMI (kg/m2)<18.5	-	13.9	15.3	22.8	15.3	-	14.7	24.2
Smoking	0.7	-	2.3	3.2	2.3	-	1.4	3.1
Currently pregnant	17.2	4.7	3.1	5.8	3.1	2.3	3.4	6.6
Less than three antenatal clinic visits	80.8	53.0	22.9	29.4	22.9	8.6	40.7	21.9
Delivery at home	46.2	50.3	12.6	39.5	12.6	17.0	54.0	50.6
Non-professional delivery assistance	43.7	47.0	7.7	31.7	7.7	7.0	27.9	41.7
No postnatal check-up on child within two days after delivery	65.4	32.9	2.0	72.3	2.0	74.1	19.2	71.9
Unsafe disposal of child's stool§	59.7	-	32.7	50.8	32.7	49.4	50.5	72.0
No exposure to media at least once a week	33.3	34.8	19.5	23.3	19.5	3.2	18.9	38.4
Not reading newspaper at least once a week	94.0	90.1	79.8	78.6	79.8	61.6	63.7	80.4
Not listening to radio at least once a week	62.1	94.6	49.3	48.1	49.3	63.1	62.0	67.2
Not watching TV at least once a week	52.1	37.0	29.8	38.5	29.8	4.1	30.4	45.8
Low overall attitude toward domestic violence	86.1	18.9	54.0	30.2	54.0	32.1	56.1	82.6
No decision on respondent's health care	53.6	25.7	8.7	54.4	8.7	11.5	16.5	6.1
No decision on large household purchases	59.5	31.2	7.8	65.7	7.8	25.1	27.8	5.8
No decision on visits to family or relatives	48.9	28.5	4.4	59.2	4.4	14.5	13.4	6.7
No decision on what to do with money husband earns	70.1	33.6	3.9	53.5	3.9	10.8	13.5	21.5
Low women empowerment	76.6	37.9	12.1	69.3	12.1	24.3	30.8	23.3
**Individual level**								
Child age of 6-11 mo	31.7	32.7	34.4	31.7	34.4	32.8	32.1	33.2
4th or lower birth order	34.7	5.2	7.0	7.9	7.0	4.9	12.4	23.4
Small perceived birth size	24.1	-	10.7	17.3	10.7	11.6	12.2	8.2
Diarrhoea	37.2	4.7	19.3	11.8	19.3	19.9	15.9	15.3
Cough	27.6	35.9	25.7	25.3	25.7	41.8	18.7	19.2
Fever	35.9	33.2	35.5	26.1	35.5	37.9	21.3	17.4
Anaemia	-	-	75.8	69.0	75.8	-	77.2	56.3
Vitamin A supplementation in the past 6 mo	47.6	72.7	62.9	70.6	62.9	77.4	49.3	61.6

BMI – body mass index

*Sampling design and weights of Demographic Health Survey of the eight countries were adjusted in the estimation of percentage using Stata syntax *svyset*.

†Improved drinking water sources included piped into dwelling/yard/plot, public tap/standpipe, tube well/borehole, protected well/spring, rainwater, and bottled water, as presented in Demographic Health Survey.

‡Improved sanitation facilities included flush to piped sewer system, flush to septic tank/pit latrine, ventilated improved pit latrine, pit latrine with slab, or composting toilet, as presented in Demographic Health Survey.

§Safe disposals included using toilet/latrine or buried.

### Minimum dietary diversity by child gender

The proportion of children who met MDD in eight countries is presented for the overall population and by child gender (**Table 2**).

**Table 2:** Weighted percentage of children 6-23 months of age meeting Minimum Dietary Diversity (MDD) in eight countries in South Asia and Southeast Asia

	Total	Male	Female	*P*-value*
	**Weighted % (95% CI)**	**Weighted % (95% CI)**	**Weighted % (95% CI)**	
Afghanistan	20.9 (18.6-23.4)	20.4 (18.0-23.1)	21.3 (18.6-24.3)	0.475
Bangladesh	41.0 (38.7-43.2)	40.4 (37.6-43.4)	41.5 (38.3-44.8)	0.605
Pakistan	13.4 (11.3-15.7)	12.7 (10.3-15.5)	14.1 (11.7-17.0)	0.304
Nepal	43.1 (40.1-46.2)	42.1 (38.5-45.8)	44.3 (39.3-49.4)	0.498
Cambodia	39.5 (36.7-42.3)	39.3 (35.4-43.2)	39.7 (35.8-43.8)	0.864
Indonesia	53.6 (51.7-55.4)	52.3 (49.9-54.8)	54.9 (52.3-57.4)	0.140
Myanmar	20.9 (18.1-24.0)	22.9 (19.0-27.4)	18.5 (15.1-22.4)	0.095
Timor-Leste	25.5 (22.6-28.6)	24.0 (20.7-27.7)	27.0 (23.2-31.1)	0.183

CI – confidence interval

*Weighted χ^2^ tests were conducted to compare MDD status between male and female children in the eight countries (all *P* > 0.05).

Indonesia had the highest proportion of male (52.3%) and female (54.9%) children meeting the MDD, while Pakistan had the lowest (male = 12.7%, female = 14.1%). There were no statistically significant gender differences in the proportion of children achieving MDD between male and female children in all eight countries (χ^2^ test, all *P* > 0.05).

**Figure 1:**
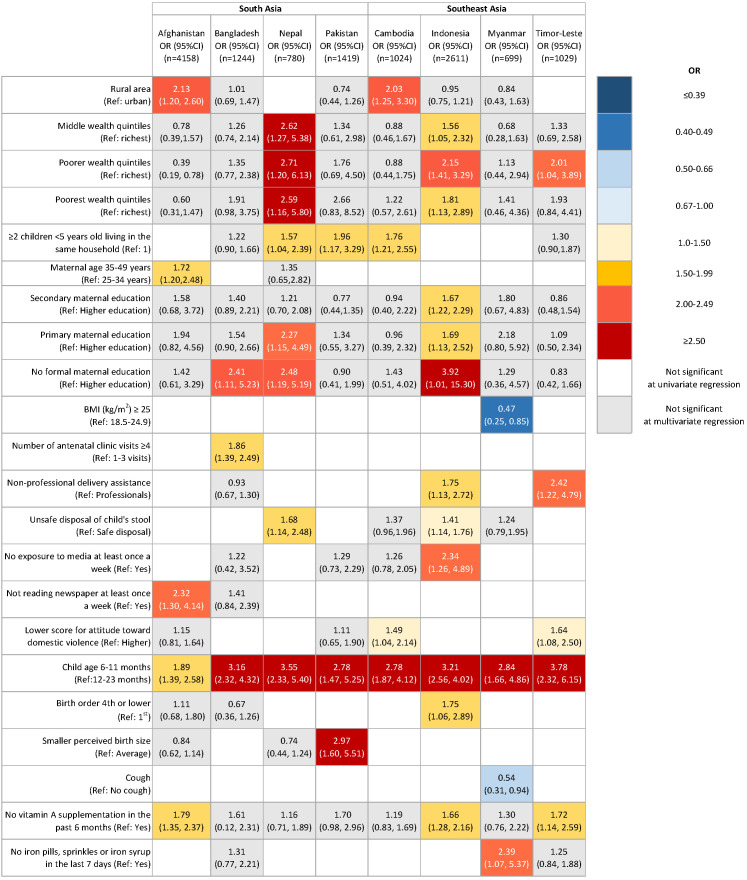
Risk factors of not meeting MDD among male children 6-23 months of age in eight countries in South Asia and Southeast Asia Abbreviation: OR, odds ratio; CI, confidence interval; BMI, body mass index; Note: Survey design and weights of Demographic and Health Survey were accounted for using Stata syntax *svyset* in the univariate and multivariable logistic regression model; Only the characteristics that had significant adjusted ORs for not meeting minimum dietary diversity in the multivariable logistic regressions in at least one country were presented in the table.

**Figure 2:**
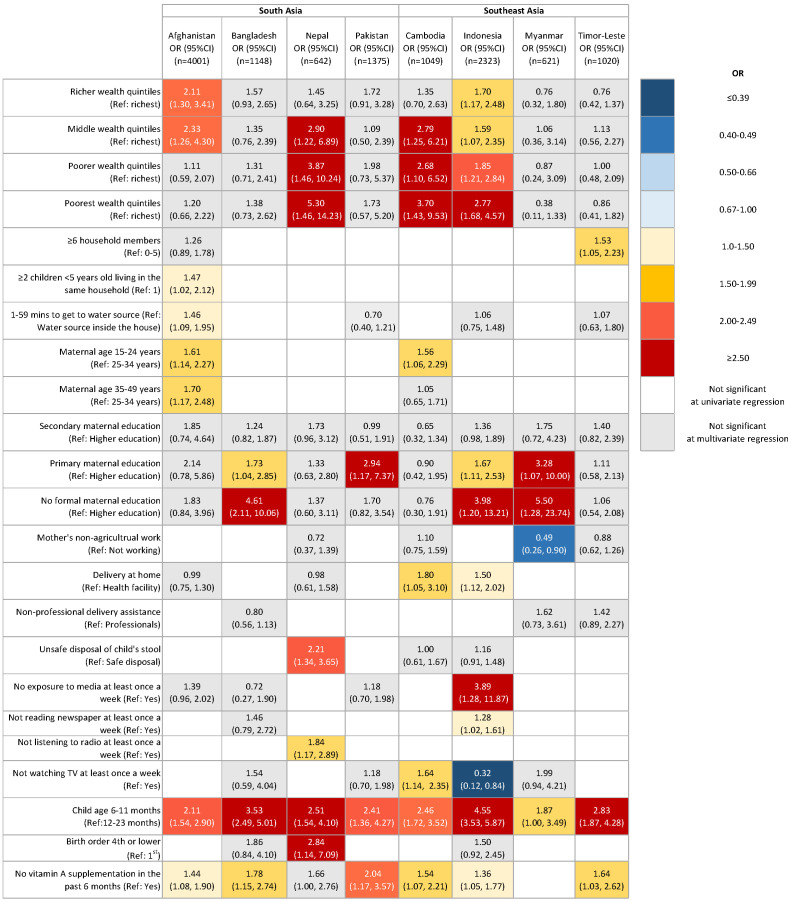
Risk factors of not meeting MDD among female children 6-23 months of age in eight countries in South Asia and Southeast Asia Abbreviation: OR, odds ratio; CI, confidence interval; BMI, body mass index; Note: Survey design and weights of Demographic and Health Survey were accounted for using Stata syntax *svyset* in the univariate and multivariable logistic regression model; Only the characteristics that had significant adjusted ORs for not meeting minimum dietary diversity in the multivariable logistic regressions in at least one country were presented in the table.

The gender-common and gender-specific risk factors for young children who did not meet MDD are shown in **Table 3**, as well as **Figure 1** and **Figure 2**.

**Table 3:** Summary of gender-common and gender-specific factors of not meeting MDD by countries among children 6-23 months of age

Country	Common*	Male-specific^†^	Female-specific^†^
Afghanistan	Child young age	Rural area	Poor wealth quintiles or more children under five years old
	No vitamin A suppl	Maternal old age	Maternal old age
		Not reading newspaper	Maternal young age
			Longer time to get to water source
Bangladesh	Child young age	Maternal poor education	No vitamin A suppl.
	Maternal poor education	Higher number of antenatal clinic visits	
Nepal	Child you age	Two or more children under five years old	No listening to radio
	Unsafe disposal of child stool	Poor mother education	Low birth order
	Poor wealth		
Pakistan	Child young age	Two or more children under five years old	Two or more children under five years old (protective)
		Small birth size	Poor mother education
			No vitamin A suppl.
Cambodia	Child young age	Rural area	Poor wealth
		Two or more children under five years old	Maternal young age
		Tolerance toward domestic violence	No watching TV
			Delivery at home
			No vitamin A suppl.
Indonesia	Child young age	Non-professional delivery	Delivery at home
	Poor wealth	Unsafe disposal of child stool	No reading newspaper
	Maternal poor education	Low birth order	No watching TV (protective)
	No exposure to media		
	No vitamin A suppl		
Myanmar	Child young age	BMI>25 kg/m^2^(protective)	Maternal poor education
		Cough(protective)	Mother’s non-agricultural work (vs no working) (protective)
		Not taking iron suppl.	
Timor-Leste	Child young age	Poor wealth	Family size (>5)
	No vitamin A suppl	Non-professional delivery	
		Tolerance toward domestic violence	

MDD – minimum dietary diversity, BMI – body mass index, suppl – supplementation

*Gender-common risk factors were the characteristics which were statistically significant in both the logistic regressions for male and female children.

†Gender-specific risk factors were the characteristics which were statistically significant either in the logistic regression for male or female children.

### Gender-common risk factors

Among all eight study countries, being younger was a factor in failing to meet MDD for both male and female children, compared to those aged 12-23 months.

The odds of not meeting MDD were associated with lower maternal education levels. Maternal informal education is related to the failure to meet MDD among male children in Bangladesh, Indonesia, and Nepal, and among female children in Bangladesh, Indonesia, and Myanmar, compared to mothers with secondary or higher education.

The odds of not meeting MDD were associated with not receiving vitamin A supplementation in the past 6 months. Among male children, the odds ratios were significantly higher in Afghanistan, Indonesia, and Timor-Leste. Among female children, the odds of not meeting MDD were significant in Pakistan, Timor-Leste, Cambodia, Afghanistan, Indonesia, and Bangladesh.

The odds of not meeting MDD increased among poorer wealth quintiles in a dose-response manner among male children in Indonesia, Nepal, and Timor-Leste, and among female children in Indonesia, Cambodia, and Nepal compared to the richest wealth quintiles.

Having two or more children aged under five years was associated with not meeting MDD among male children in Cambodia, Nepal, and Pakistan, compared to having only one child. Among female children, an association with two or more children showed mixed direction with not achieving MDD in Afghanistan and in Pakistan.

### Gender-specific risk factors of male children

The risk of not meeting MDD differed by the place residence (urban vs rural) in Afghanistan and Cambodia. Compared to safe disposal of children’s stool, unsafe disposal practices were a risk factor for not meeting MDD among male children only in Indonesia and Nepal.

In South Asian countries, older maternal age (35-49 years), compared to 24-34 years, was a significant risk factor for not meeting MDD among male children in Afghanistan. The odds of not achieving MDD were 2.32 times higher among Afghanistan mothers who did not read the newspaper at least once a week, compared to mothers reading the newspaper. Having four or more times of antenatal visits was associated with male children’s not meeting MDD in Bangladesh.

Among Southeast Asian countries, having non-professional delivery assistance was associated with failure to meet MDD among male children in Indonesia and Timor-Leste, compared to having professional delivery assistance. The odds of tolerance for domestic violence were significantly associated with not meeting MDD in Cambodia and Timor-Leste. Notably, maternal BMI≥25.0 was a protective factor for meeting MDD in Myanmar. The odds of not meeting MDD were higher among women who were not exposed to media at least once a week, compared to mothers exposed to media in Indonesia. In Myanmar alone, not taking iron pills/sprinkles with iron or iron syrup in the last seven days was a factor for not meeting MDD.

### Gender-specific risk factors of female children

Maternal age was also a risk factor for not meeting MDD. In Afghanistan, mothers aged 15-24 years or 35-49 years, compared to 25-34 years old, showed a higher risk of not meeting MDD among their female children. In Cambodia, female children of mothers aged 15-24 years were less likely to meet MDD.

While in Cambodia, where mothers not watching TV at least once a week was a risk factor for not meeting MDD, the same variable became a positive factor for meeting it in Indonesia. In Cambodia and Indonesia, at-home child delivery was a risk factor for children being less likely to meet MDD compared to delivering their children in a health facility.

## DISCUSSION

This study examined the gender-common and gender-specific factors of not meeting the MDD among children aged 6-23 months in eight South and Southeast Asian countries.

### Gender-common risk factors

Younger age (6-11 months), compared to 12-23 months, was a factor in not meeting MDD among both male and female children. Children aged 6-11 months are mostly breastfed, while also being fed some limited food resources and types, depending on their adaptation to family foods [20,21,39]. As the child gets older, parents feed their child various foods [40].

Not receiving vitamin A supplementation was associated with failure to meet MDD. Children who do not receive vitamin A supplementation could have limited access to public health services. For example, these children may not be provided with immunization services in a timely manner by local health providers [21], or nutrition services that provide vitamin A supplementation might not be accessible due to lack of transportation.

Maternal education is an important factor affecting dietary diversity for both male and female children. Well-educated mothers were more likely to access information and educational messages through antenatal and postnatal services or various communication channels about nutrition, thus preventing traditional misbelief about feeding practices [21,41-44].

Poor wealth quintiles were a risk factor for young children not meeting MDD because these households were less likely to have food secure and afford multiple types of nutritious food from the local market [45]. Households with higher economic status were more likely to consume nutrient-rich foods (eg, eggs, meat, milk), which might increase their diet diversity [41].

In addition, households with more than two children under five years old were associated with not meeting the children’s MDD, indicating increased competition among siblings for food consumption and childcare [7,20,24,39]. Having many young children might place more pressure on food consumption and make it more challenging to provide all children in the household with sufficient food [46].

### Gender-specific risk factors for male children

Male children whose mothers received professional delivery assistance were more likely to meet MDD. A study carried out in Indonesia showed that children delivered by caesarean section with professionally trained birth attendants were more likely to meet MDD [47]. A possible reason could be that delivery by caesarean section is associated with early cessation of breastfeeding [48].

Among male children, mothers’ safe disposal of their children’s stool indicated a higher chance of MDD being achieved, compared to those who had unsafe disposal. Open defecation or other unsafe stool disposal practices could increase the risk of diarrheal disease and affect the children’s nutrient absorption [49].

A mother’s tolerant attitude toward violence meant that MDD among children was less likely to be achieved. Those children can be negatively influenced by the mothers’ exposure to domestic violence [50]. The negative psychological outcome caused by domestic violence, such as depression, could lead to a weaker mother-child bond, which would decrease the quality of feeding practice [51].

Households being located in rural areas was another factor related to male children not meeting MDD. It is possible that, due to the local market being inaccessible to the household, it would be difficult for mothers to have diverse or equitable knowledge beyond local traditions [42,52,53].

### Gender-specific risk factors for female children

Mothers not watching TV and mothers being less exposed to media were risk factors for not achieving MDD in female children. In a culture where males were preferred, females might have less access to media and TV, especially in poor households, due to media and TV subscriptions requiring additional expenses [52,54]. In Cambodia, where biomass is more frequently used as an energy source compared to electricity [55], the lack of access to media could prevent mothers from acquiring professional information and new knowledge by watching TV.

Mothers delivering their children at home was one of the risk factors for female children not meeting MDD. Households living in rural patriarchal society do not have sufficient access or ability to receive information for feeding practices [56].

The younger age of mothers might be related to more school dropouts and inappropriate feeding processes of their children [57]. Therefore, mothers who gave birth at a young age might lack professional knowledge of age-appropriate feeding [58].

### Limitations

This present study has some limitations that should be considered. First, the child dietary diversity scores were based on the responses from the mothers’ 24-hour recall in the DHS survey, which might not accurately reveal the usual children’s dietary behaviour patterns. Mothers were not asked to report the amount of food items consumed. Second, some variables that are essential for determining a child’s food consumption (ie, food security, paternal-related factors, food expenditure) were not available in the DHS survey. Third, our analysis model did not account for within-country seasonality of food availability. Types of affordable food and diversity of market-available food are season-dependent [59]. To alleviate the seasonal impacts on child’s food intake caused by various data collection periods between countries, we built a unique analysis model for each country. However, in some countries, the survey was administered through a relatively long period. The influence of being interviewed in different seasons within a country remains a limitation.

### Recommendations

Among both South and Southeast Asia regions, younger age (6-11 months) and not receiving Vitamin A supplementation in the past six months, maternal education levels, and household wealth quintiles were risk factors found in both male and female children for not meeting MDD. In both regions, gender equity in Vitamin A supplementation is expected to promote MDD. For example, depending on the differences on how males and females process information, approaches to raising the awareness about the gender equity in Vitamin A supplementation for parents can be tailored to meet the nutritional needs of all children [60].

Both having unsafe disposal of children’s stool and living in the rural areas were risk factors for male children not achieving MDD in both South and Southeast Asia. Political leaders and policymakers should put more effort into strengthening infrastructure and development in rural areas to enhance the accessibility of local markets, professional health information, and sanitary practical training for community households. To improve awareness toward professional delivery and domestic violence, future programs should also incorporate actions to raise gender equality awareness among parents, to focus on the existing gender gap in MDD for young children.

Meanwhile, mothers delivering at a young age was the only risk factor found in both South and Southeast Asian regions among female children. We should consider multiple ways to raise the awareness of the impact of delivery at the proper age while considering socio-economic status, traditions, agency, and parents’ beliefs. Policymakers must advocate for gender equity in the education system and the job market to prevent women from quitting school at a young age and facing gender-based stereotyping and discrimination [61].

## CONCLUSIONS

By analysing the DHS survey from eight countries in both South and Southeast Asia, this study revealed that gendered differences among factors for not meeting MDD exist. These findings can inform program planning and research in child nutrition to identify populations at risk and modifiable risk factors. Meanwhile, this study’s findings also indicated that it is worthwhile for future policies and programs to consider employing gender-specific approaches regarding the improvement in child nutrition.

## Additional material


Online Suplementary Document
