# Supplementary figures and images for: Gender-common and gender-specific determinants of child dietary diversity in eight Asia Pacific countries
Source: J Glob Health. 2022 Oct 1;12:04058. doi: 10.7189/jogh.12.04058 (PMC9526379; doi:10.7189/jogh.12.04058)

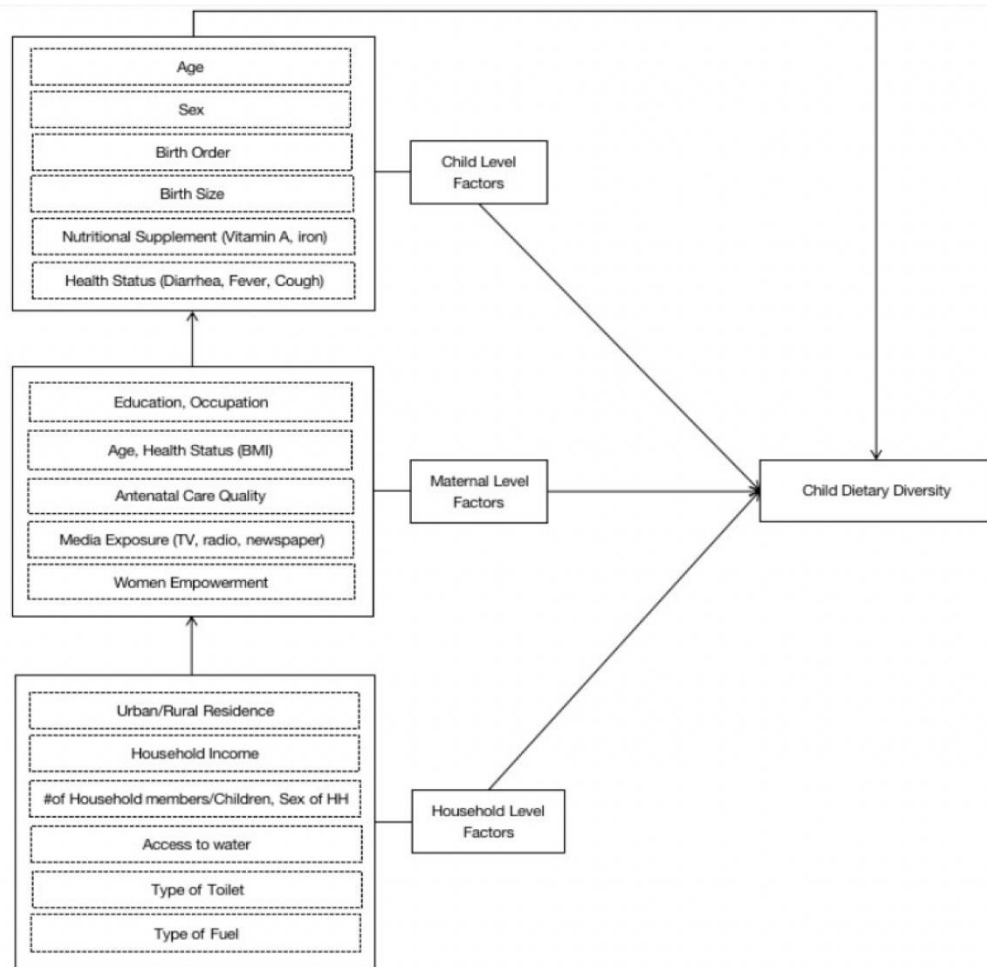

Figure S1. Conceptual Framework of Child Dietary Diversity.

Supplement: Online Suplementary Document [file jogh-12-04058-s001.pdf]
